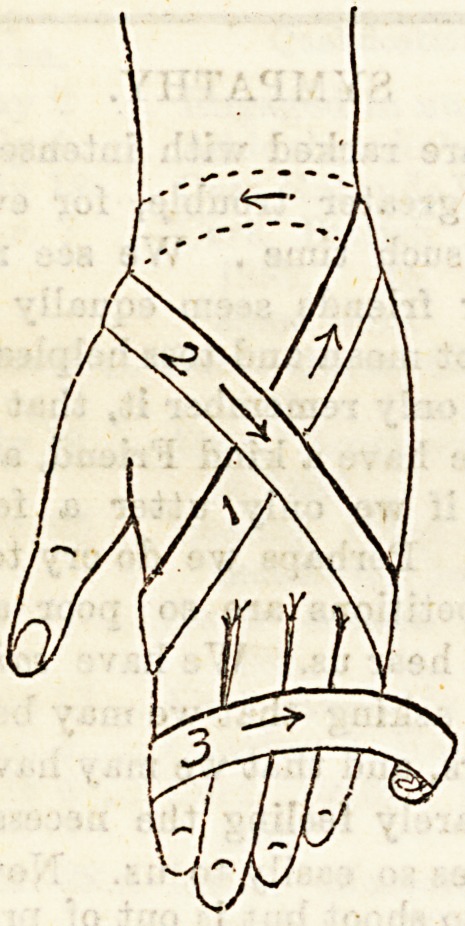# The Hospital Nursing Supplement

**Published:** 1891-07-25

**Authors:** 


### "The Hospital" Nursing Mirror.

Being the Extra Nursing Supplement of "The Hospital" Newspaper.

Contributions for this Supplement should be addressed to the Editor, The Hospital, 140, Strand, London, W.C., and should have the word "Nursing" plainly written in left-hand top corner of the envelope.

### En Passant.

ZENANA WORK.—The Indian Instruction Society and Zenana Mission, of Adelphi Terrace, have sent us a sheaf of booklets descriptive of their work. "Healed and Saved" is a story of the hospital at Lucknow, and "Hindu Widows" is a picture of a death in life it is terrible to contemplate. The chief agents employed by the society are fully-qualified medical women and trained native nurses; by healing the body these missionaries try to reach the soul, and to somewhat lift the veil of ignorance which blights the life in a zenana.

SHORT ITEMS.—This week's list of contributors to the Princess of Wales' Fund for Mrs. Grimwood will be found on page 196.—Miss Sleeman, Miss Webly, and Miss Blennerhassett have arrived at Fort Salisbury, Mashonaland.—The Rev. H. Pelham Stokes, vicar of Isleworth, appeals for funds to send trained nurses to Palestine, to attend to the influx of poor sick Jews now returning to the Holy Land.—Miss Frances Close, late of Newcastle, has started a Private Nursing Institution at St. Leonards-on-Sea.—Miss Champion has opened a Private Home for patients at Woodside, Plymouth.—South Cork Infirmary will, in future, be supplied with nurses from Miss Woodruff's Institute, instead of from the City of Dublin Hospital.

ASYLUM ATTENDANTS.—The Commissioners in Lunacy have issued their annual report, and they make special mention of the necessity for care in selecting and training attendants. Such persons, they say, must be qualified not only by knowledge, but by personal temperament; for lunatics are apt to try the temper of attendants, who should, therefore, be persons capable of habitual self restraint. To retain such attendants as are desired, adequate remuneration must be given. If they were rewarded with salary or wages only without prospect of pension, a higher scale would, it is considered, be necessary, but the Commissioners declare that fair salaries or wages, with the prospect of liberal pensions after disablement or reasonable length of service, offer the most influential inducements to really suitable persons to enter asylum service, and to remain in it as a permanent occupation.

HOLIDAY HINTS. — Yet another suggestion for a party of three nurses; Miss Ferry, of Woodland House, Matlock Bath, Derbyshire, would receive them for 30s. a week for the three; the house is near Chatsworth and Dovedale, and all the beautiful Peak country. Miss Ferry was once Matron at Tenbury, and has always been good to nurses. Surely no pleasure-seekers ever so thoroughly enjoy their holidays as nurses do; the unusual freedom from institution life, the leaving behind of sad scenes, and the glorious feeling that the holiday has been honestly earned, all add to the joys of the days of idleness. Put aside all cares and fears for a few days, ye workers in a noble cause, and gazing at the beauties of nature, and drinking in her peace, acknowledge that the world is not all sin and suffering. It is right to enjoy life; it is right to rest at times from our labours; therefore we would beg all nurses to take without scruple all the pleasures that fate may provide for them during their brief holidays. Then, the rest done, they shall shoulder arms once more, and march forth strengthened and refreshed.

STOCKTON NURSING ASSOCIATION.—On the last day of June a meeting of the supporters of this association was held, Mr. Cradock, J.P., presiding. Mr. Makepeace read the report, which stated that in accordance with the resolution passed at a public meeting held on April 24th last the members of the two provisional committees appointed to formulate a scheme for providing trained nurses to attend the sick poor in the Parliamentary borough of Stockton-on-Tees, and to collect subscriptions and donations in aid of the same, met for the first time on the 1st day of May, and have subsequently met on seven different occasions. Three trained nurses have been engaged; £425 has been received in subscriptions and donations, and the work has been got into going order. Two important questions are yet to be decided: Shall the Association affiliate with the Jubilee Institute, and shall it build a home for the nurses? The Provisional Committee leave these and other points to the newly-elected Executive Committee, of which Lady Londonderry is the President.

THE HEBRIDES.—Those of us going to the Highlands for our holidays may dream that we are going without the region known to nurses; but this is not so, as the following notes from the Lady Superintendent of the Glasgow Sick Poor Association show: "In the spring in the *Glasgow Herald*, and in June in the *Lancet*, there were accounts of the 'Fever Scare at Mull.' Consequently, two of our nurses were sent, one being a midwife. One of the patients gave birth to a baby, with the rash thick upon it. The nurses returned last week, after having done everything for their patient, washing, cooking, &c.; and not a death has occurred since they were in attendance. Last week one nurse returned from St. Kilda, one of the Western Islands, sixty miles from mainland, and is cut off from all communications for ten months of the year. There were four births; this time all the mothers lived, but three babies died of the usual complaint—tetanus. It is noticed if the babies vomit they live. The symptoms occur as soon as the cord drops off, then the child begins to fret and cry, when suddenly it becomes convulsed. These fits occur every twenty minutes for twenty-four hours, then the infant dies. The midwife returns this week."

SICK PAY.—When a hospital nurse a few years ago went round the wards and got a list of those male patients who needed certificates in order to draw their sick pay from their clubs how little she thought that one day she also could secure pay in time of sickness. This is the part of the Pension Fund which has been of most use, as yet; and many are the letters of thanks written to those who promoted it. Here is a specimen: "I specially have cause to thank you for what you have done, for during my long illness I have been spared the added pain of being a heavy expense to my mother, whose income could ill have borne it, though at the same time I cannot help being sorry to be such a tax on the Fund." There is no necessity whatever, though, to be sorry for the Fund; it is the most cheerful and the most successful Fund possible; started on a business basis it has no need to fear. Had the howls of the unsatisfied been heeded at first, and the Fund started with very low premiums, with a sort of chance and hope that nurses wouldn't be ill, then it might have become a sad and stricken Fund. But the City merchants were not such fools as not to know that "business is business," and they look forward with a smile to the time, twenty years hence, when some thousands of nurses may be living in comfortable retirement on their allowances. And in the meantime hundreds of nurses appreciate the sick pay,

### Protecting the Public.

In his evidence before the Lords' Committee, as reported in last Monday's *Times*, Dr. Bedford Fenwick said: "The registration only affected women who were trained. It was true that the public had not adequate protection by the mere granting of a certificate, and the object of the register was to afford to the public some such protection. The Registration Board only issued certificates to nurses who could prove that they were trained and of good character, and they paid 10s. 6d. each for the privilege."

After these statements there is no choice left to us save to give specimens of the nurses on this register, that the public may judge for themselves the meed of protection they are likely to derive from it. We did not wish to bring forward the names and histories of the nurses unfavourably known to us who are on the register; we have taken trouble to collect much information on the subject, but our sympathy is with the nurses, and we shall make as little comment as possible. The following are extracts from the register:—

| Name. | Date of Registration. | Qualification, Training, &c. |
|---|---|---|
| A., S. ... | 1890, May 2 ... | Engaged in nursing before 1870. |
| B., M. ... | 1890, July 4 ... | Addenbrooke's Hosp., 1886—7. |
| B., C. E. | 1890, July 18 ... | Cert. Rotunda Hosp., Dublin, 1888. |
| B., R. S. | 1890, Mar. 7 ... | Cert. British Lying-in Hosp. in 1882. In professional work since 1868. |
| B., A. ... | 1890, Mar. 7 ... | Cert. Queen Charlotte's Hosp. in 1885. |
| B., A. ... | 1890, July 18 ... | Royal Southern Hosp., Liverpool, in 1883. |
| B., K. S. | 1890, Nov. 25 ... | London Hosp., 1887—8. Weymouth St. Inst., 1888—9. |
| B., E. ... | 1890, Mar. 7 ... | Cert. British Lying-in Hosp. in 1881. |
| C., E. ... | 1890, June 13 ... | Cert. Adelaide Hosp. |
| C., E. R. | 1890, Mar. 7 ... | Charing X Hosp. in 1877. |
| C., H. ... | 1890, July 4 ... | Cert. British Lying-in Hosp. in 1885. |
| D., M. P. | 1890, Mar. 28... | Miller Memorial Hosp., Greenwich, in 1885. |
| H., E. ... | 1890, May 2 ... | Monthly nursing since 1883. |
| H., E. ... | 1890, Mar. 7 ... | London Hosp. in 1885. |
| L., S. ... | 1890, June 13 ... | Parish and sick nurse, Prittlewell, 1884—88, and Slough, 1888 to date. |
| M., F. ... | 1890, July 18 ... | York Asylum. |
| N., M. ... | 1890, May 2 ... | London Hosp. in 1889. |
| O., H. H. | 1890, May 2 ... | Trained under midwife in 1867. |
| W., R.... | 1890, July 18 ... | In professional work before 1870. |

We should like to know what the public think of the above "qualifications." taking into consideration the assertion of Dr. Bedford Fenwick that "The Registration Board only issue certificates to nurses who could prove that they were trained and of good character." What training, pray, had F. M., against whose name stands a brief and undated "York Asylum"? Doubtless many of the above are worthy women, but to call them "Trained Nurses," if the above are their sole qualifications, and say that the register bearing these names is for the protection of the public, is monstrous. The public can have untrained nurses if it likes, but if it wants a trained nurse it had better demand one holding the certificate of a hospital and not the certificate of the British Nurses' Association. We would gladly not touch on the question of the character of the nurses on the register, but we also have some thought for the public. In The Hospital for April 11th we pointed out that there was on the first page a nurse who had to leave her training school for having in her possession an article belonging to another probationer; we do not wish to give names, or go into details on these subjects; however, there is one case so public it can do no harm to refer to it. In her evidence before the Lords' Committee, in answer to question 6,510, the Matron of the London Hospital stated: "E. H. proved a very unsatisfactory probationer. . . . Nothing could have developed her into a good nurse." We believe E. H. was for about five months at the London Hospital, and left with the above character, and yet she is on the register of *trained* nurses. It is generally acknowledged that a year in a good hospital, or three years' experience, is the lowest standard at which the name of nurse should be given. We would point out that there appear to be nurses on the register who have had neither training nor three years' experience, and who are, nevertheless, now backed up by certificates signed by the Princess Christian; this, Dr. B. would have us believe, is done "for the protection of the public."

### Everybody's Opinion.

### FOR THE LEPERS.

Miss Gethen, 55, Burton Crescent, W.C., writes: In acknowledging the box of scrap and picture books sent last Christmas by some of your readers to the children in the Leper Hospital at Colombo, the Chaplain says "I see so much of their sufferings and misery that I am most grateful to those who help to make their lives more tolerable. They are much pleased with the albums and books, and the merest trifles serve to amuse children whose lives are so dull, monotonous, and painful. . . . I trust you will forgive me if I add that anything more you or your friends may send will be most gratefully accepted and acknowledged." If any of your readers are willing to send gifts, however small, I shall be glad to receive and forward them in a box, which I hope to have filled by the 1st November with Christmas presents for these afflicted children.

### Examination Questions.

The London Hospital. July 2nd, 1891, seven p.m. to ten p.m. Full marks 60—10 for each answer. The first three questions must be answered to ensure "passing":—

I.—A serious operation (ovariotomy) has to be performed in a private house. (1) How should the patient be prepared? (2) What kind of bed and bedding should be selected? (3) What position should the bed occupy in the room? (4) What preparation should be made for the operation itself? (5) What dressings are usually required in such a case? (6) What points in the patient's condition must be specially noted during the first 24 hours?

II.—What is meant by (1) hydrocarbon diet and (2) diabetic diet? Explain something of the principles on which these diet tables are constructed in relation to the diseases for which they are prescribed.

III.—How would you make and change the bed of a helpless patient? What precautions should you take to prevent a bed sore? How should you treat one when existing?

IV.—What happens when we breathe—(1) To the chest; (2) to the air breathed; and (3) to the blood in the lungs? Mention some of the results of defective ventilation, and discuss any simple methods for securing safe and efficient ventilation in a small sick room.

V.—What are the chief antiseptics you have seen used, and what should be their strength in the following cases: (1) For washing a wound; (2) for cleansing instruments; (3) for disinfecting excreta and discharges?

VI.—What points are important in the preparation and administration of the different kinds of enemata? State any details a nurse should bear in mind in connection with the various methods of administering drugs.

### Asylum Articles.

### IV.—COMPARISONS.

Of course they are odious, but then they are also instructive; and the one chief comparison I would draw between different asylums is with regard to the freedom with which the outside public is admitted. At Berrywood I was permitted not only to go wherever I liked, but also to sit and work amongst the patients, and on one occasion I dined with them. If the public is excluded from a building it inevitably imagines all sorts of horrors are conducted within the closed gates; most of the terrible stories about asylums arise not from facts but from imagination dwelling on what is not allowed to be known. On my last day at Berrywood a nice, quiet-looking girl came to me and said she was illegally detained there through the machinations of her mother, and would I put her case before the law courts? I answered simply that I would speak to the medical officer, for I had seen throughout how excellent were the arrangements and knew well that the patients were free to write to whom they would, to see their friends, and to see the Commissioners; all this apart from the many officers of the institution. It was therefore absolutely impossible that any sane person should be detained there. But some years ago, going round an asylum managed on the secretive system, a patient slipped a note of similar import into my hand. The medical officer demanded the note, and after reading it, I gave it him, and I was hurried out of the ward. Well! for a long time that first incident rankled in my mind, simply because investigation was not courted, and explanations were not given. The following letter from the medical officer of an asylum in North London is an odious comparison with the hearty invitation which took me to Berrywood:—"I should be glad to show you round the wards, but it is out of my power to do so. I would require to get the consent of the committee, and it is quite possible they might refuse."

For another comparison look on the above illustration, and then picture in your mind the ordinary attendant in a shabby black gown, and with no sign of her office save the jailor-like bunch of keys at her belt. Surely it is better that the attendant should wear a uniform something like the above, which is a sketch of that worn at Berrywood. The dress is dark blue; cuffs, collar, and soft silk crown to cap are scarlet; the braiding is gold; the whole effect is very military and smart.

### SYMPATHY.

When our bodies are racked with intense pain, our minds are often in still greater trouble, for every little worry appears greater at such time. We see no way out of our difficulties, and our friends seem equally powerless to help us. But we need not moan and toss helplessly on our couch; we know, if we can only remember it, that we are never left to ourselves, that we have a kind Friend, a Great Physician, who will help us if we only utter a few short broken sentences of prayer. Perhaps we do cry to God in our extremity, but our petitions are so poor and weak that we hardly hope He will hear us. We have *said* our prayers in health—sometimes; asking that we may be preserved in all dangers and disasters, and that we may have our daily bread given us, though barely feeling the necessity of asking for what generally comes so easily to us. Now we are like an archer who wishes to shoot but is out of practice. We send up a few arrows of prayer so feebly at first that they appear to fall back to earth and to be lost. Yet it is not so, our heavenly Father sees our poor efforts, and answers them by the gift of perseverance; as we try again, and yet again, our aim becomes better, our grasp is stronger, we bend the bow to its full width, and our shaft speeds straight to the golden centre of the throne of Grace, and is answered in full measure. And the help and strength we receive comes from a God full of love and sympathy for His suffering children. He gives us patience to bear our woes, and stirs up our brethren to aid us. In this Christian country of England there are thousands of good men and women whose love and sympathy are poured without stint on those who cannot help themselves. Their love to God teaches them to love their neighbours. They give lavishly from their purses, they visit the sick and needy in their affliction; with their hands they minister to bodily wants, with their hearts they beseech the Almighty to have, for the suffering, mercy on both souls and bodies. The hearty, earnest prayer of a righteous man availeth much. Rejoice, dear friends, even in your sickness, for you have a Saviour and Protector, who watches over all mankind, and causes the sun to shine, and the rain to fall alike upon the evil and the good.

"Thou art as much His care, as if beside
Nor man nor angel lived in heaven or earth."

We may rest in this thought and in the promise, "I will never fail thee nor forsake thee."

"When weary ones beneath their burden sigh,
And wish the day were gone, then of Thy Love
Let angels come from Thy dear rest above,
And take away the veil that they may see
A pure white sunbeam shining down from Thee."

### Lectures on Surgical Ward Work and Nursing.

By Alexander Miles, M.B. (Edin.), C.M., F.R.C.S.E.

### Lecture XXXI.—BANDAGES FOR UPPER EXTREMITY.

Bandage for Thumb is simply a figure of eight, the turns going alternately round the ball of the thumb and the wrist till the whole of the former is covered in.

Bandage for Fingers.—It is rarely necessary to bandage each finger separately. In doing so, however, the ordinary spiral bandage is employed, the end being fixed by a figure of eight turn round the wrist. When all the fingers require covering in it is better to pad them carefully, and apply a single bandage to support all of them together, than to apply a bandage to each individually.

To Bandage the Hand and Forearm, place the limb in the position of pronation, that is with the palm turned towards the ground, so that it corresponds in position to the sole of the foot. The other parts of the upper extremity will then correspond to those of the lower. Thus the hand represents the foot, the wrist the ankle, and the forearm the leg. The forefinger corresponds to the great toe, the little finger to the little toe, and the thumb to the heel. Applying the general rules, and employing the appropriate form of bandage, the hand and forearm are covered in exactly the same way as the foot and leg were. The thumb is left free.

As in the lower extremity, so here the *whole limb* may be covered in by continuing the above bandage in the form of a figure of eight over the elbow, of a simple or reversed spiral as may be necessary over the upper arm, and finishing with a spica round the chest.

To Bandage a Closed Fist, as for example in treating fracture of the metacarpal bones by means of a pad in the palm of the hand, the best means is by a series of figure of eight turns. The hand being closed and pronated, a fixing turn is made round the wrist, and then a series of figure of eight loops are applied alternately round the wrist and the hand, passing from the little finger towards the index. To finish off the bandage a turn is made circularly round the hand and this catches in all loose pockets.

Bandages for Elbow.—The most convenient form of bandage to employ here is the spica, which may be either *convergent* or *divergent*. The former is simply a figure of eight, the turns converging towards the tip of the elbow; the latter is applied in exactly the same way as the corresponding bandage for the knee. Begin over the internal condyle, making the first turn cover in the tip of the olecranon process. The pockets left above and below are disposed of by the succeeding diverging turns. Such bandages are used to keep the elbow joint at rest after operations or injuries, the converging spica being specially useful in cases of fractured olecranon, as it tends to approximate the fragments to one another.

Bandage for the Shoulder.—Again the spica is used, the turns being round the arm on the one hand, and the *thorax* on the other. It is not permissible to put the second turn of the spica round the neck in place of the chest, because the movements of the patient's head inevitably relax these turns and the bandage becomes inefficient. To apply this bandage continue that of the upper arm as far as the axilla, then pass over the shoulder from within outwards, across the back into the opposite axilla, thence across the front of the chest and round the shoulder to the point of starting. By three or four such turns the whole shoulder may be covered in. This, then, is evidently an ascending spica. The descending spica is rarely, if ever, indicated in the region of the shoulder.

Handkerchief Bandages for Upper Extremity.—*For the Hand*, a useful temporary bandage to retain a dressing on the *palm of the hand* is obtained by folding a triangular bandage *en cravatte*, laying the centre over the palm, carrying the ends across the back and then round the wrist, on the back of which they are tied. This obviously consists of a double figure of eight. This bandage reversed would retain a dressing on the *back of the hand*. The *whole hand* may be covered in the same way as the foot. *For the elbow*, the same method is adopted as for the knee. *For the shoulder*, as for the hip, two bandages are required. The extra one is passed either round the neck, or across the chest, passing under the opposite axilla.

Slings.—These are used to support different parts of the upper extremity, and are in the form of triangular handkerchiefs. In applying a sling the base of the triangle is placed towards the part to be supported, *e.g.*, the elbow, or the wrist, as the case may be. The ends are carried across the shoulders, either directly or crossed, and fixed by a reef knot. They alone bear the weight, the apex being folded up neatly and fixed by a safety pin.

To Bandage a Stump.—Fix on the dressing by means of spiral turns, and then to cover in the end employ a series of divergent folds over the face of the stump, these being secured by a second set of spiral turns. *A handkerchief* may be used for this purpose, as for almost any other.

The Many-tailed Bandage of Scultetus is used when frequent dressing of a part is necessary, and where it is at the same time undesirable to disturb the limb. It consists of a firm backbone, to which are sewn at right angles a number (16—20) of shorter pieces. These are made to overlap one another for two-thirds of their width, and are long enough to encircle the limb once and half. The backbone is placed along the posterior aspect of the limb, the dressing applied, and the lowest turn folded into position, and successively all the others. The last turn is fixed by safety pins. As often as is necessary the turns may be unfolded, and the dressing re-applied without the limb being in any way moved.

### Trained Nurses' Club,

12, Buckingham Street, Strand, close to Charing Cross.

Nurses belonging to the Pension Fund are cordially invited to use the rooms of the above club on Friday, 24th, between the hours of three and ten, and on Saturday from ten a.m. till seven. Cloaks and bonnets may be left, friends can meet, nurses can wait for trains, &c. Tea can be obtained. Secretary, Mrs. Nichol.

### Notes from Australia.

MENTION was made of a man killing his wife and their four children, and endeavouring to kill himself, in a recent number of THE HOSPITAL. Much excitement as to his sanity resulted in his respite for a few days, and then the fulfilment of his death sentence. Another murderer, Wm. Coleston's trial is delayed that he may be observed as to his sanity!

Melbourne Hospital is to the fore again in disputes on deaths from chloroform, and also as to the election of its doctors' staff. Much electioneering is going on in preparation for the decision which is to be made in August. An opinion is abroad that the more money you spend the more likely you are to succeed, which means "bribery and corruption."

The Charities Commission still go a head, and if they succeed in getting some of the old and helpless taken better care of, and *not* sent to gaol as at present, there being no other place for them, one blot from Melbourne of to-day will be removed.

We certainly do peculiar things now and then in this colony. Here is or was the pretty town of Sandhurst got uneasy with its name, and by a ruse a minority succeeded in getting it changed to Bendigo, after the pugilist of that name.

Then we have had a Ladies' temperance gathering, where they roundly abused the use of tea, and then went in for women's suffrage.

Greek is no longer to be a thorn in the flesh of medical students at the University. Hitherto, no student has been able to take a medical degree unless he has first passed in Greek at matriculation. The medical faculty some time ago agreed that the smattering of the Greek language which the student thus obtained was not of the slightest assistance to him in his course, and that a knowledge of French or German —the latter for choice—would be of infinitely greater value. Upon the recommendation of the faculty the University Council proposed that a medical student should be allowed to take Greek, French, or German at his option, and this was ratified by the Senate at its meeting yesterday.

Dr. Syme has drawn up a course of theoretical teaching for the nurses of the Melbourne Hospital, and it will be proceeded with at once. At the Women's Hospital there has been much ill-feeling because two nurses were refused certificates after six months' service. A letter was read from Dr. Felix Meyer, writing on behalf of himself and Drs. Adam and O'Sullivan, honorary surgeons to the midwifery department, with reference to the two nurses who had not passed their examination and were rejected. The doctors pointed out that the girls, who had shown a deficiency in the theoretical part, were not finally rejected, but were ordered to come up again in a fortnight, thus giving them an opportunity for reading up.

An article on nurses has appeared in the *Argus*. The writer says: "What is needed is a central board of examiners, composed of medical men of known standing in the various branches of medicine, to whom should be committed the whole responsibility of all examinations for nurses in the hospitals in Victoria. What is necessary is that the status of nursing as a profession should be raised, and the appointment of a central board of examiners ought greatly to help towards this desirable end. Would it not be well that this idea should be ventilated and considered, before too many separate schemes for the training of nurses are embarked upon?" This sounds nice in print but the writer is no nurse, no, nor even a woman; only a man's mind could thus look on the purely theoretical side of nursing as of sole importance. Some of the hard, careless women who act as nurses here, would not be improved, but quite the opposite, by possessing a piece of parchment which enabled them to say they belonged to a profession; they charge quite enough and give themselves airs enough without that, and what is wanted is that they should be trained in the sweet serviceable spirit of Florence Nightingale, as well as in medical details. Training in England was begun under Miss Nightingale, and side by side with the lectures given by the doctors, run the bedside instructions in servitude and gentleness given by the Sisters. No central board can secure us better nurses here, but the radical improvement of the nursing system of the hospitals can, and will. Only those who have trained a nurse, that is, the medical officers and matrons of the different hospitals, know when a probationer can be trusted to take sole charge of a case. A central board could say, "this woman knows what should be done in a case of typhoid"; but only those who had personal knowledge could say, "this woman will do what should be done in a case of typhoid." No matter how clever a nurse is, if she is careless she is useless. The authorities of the Melbourne hospitals would do well to see that no central board is appointed to take their due authority out of their hands. Dr. Henry moved the following at the last meeting of the British Medical Association, Victoria Branch: "That in view of the accession of legally-qualified women to the medical profession in this colony, and bearing in mind the probability that in the future the ranks of the profession will every year be recruited by women medical graduates of the University of Melbourne, and, moreover, considering that a woman has been elected a member of the British Medical Association in England, I give notice that at the next ordinary meeting of the Branch I shall move that duly-qualified women are eligible as members of the Victorian Branch of the British Medical Association."

### Notes and Queries.

### Queries.

(23) Can anyone tell me of a home or hospital where a young man suffering from incurable spinal and lung disease would be admitted?—*E. J. G.*

(24) Can any nurse tell me of lodgings in Yarmouth for two nurses?—*B. B.*

(25) Is it necessary to re-fill water beds? I have had one in use for three months, ought I to empty it?

### Answers.

*An Old St. John's Nurse.*—We will make further enquiries, and will print part of your letter next week.

(20) "A Manual for Midwives," by Fancourt Barnes, can be obtained of Bimpton, bookseller, 126, Wardour Street, Oxford Street, W. (very good indeed), with illustrations.

(20) "A Manual of Midwifery for Midwives," by Fancourt Barnes, price 6s., is the best cheap book on the subject. "A Treatise on the Science and Practice of Midwifery," by W. S. Playfair, in two volumes, price, I believe, 25s. Both books have numerous illustrations.

(20) *Overton.*—The library of the Midwives' Institute, 12, Buckingham Street, Strand, contains numerous manuals on midwifery, and the Secretary could give all information as to the best manual for Overton to buy.

(21) Miss Pratt is glad to have a chance of recommending Miss Bird's apartments, at 35, Adelaide Gardens, West Cliff, Ramsgate.

(21) Apply to Miss Lime, 30, Nelson Road, Great Yarmouth. Very comfortable, and, I believe, cheap.—*R. Webster.*

*E. von de G.*—It is our duty to report all nursing cases, and we are in no way responsible for the position in which you find yourself.

*Nurse.*—Any bookseller would precure you "Culiingworth's Manual," if you told him the name of the publisher. If you choose to write to the publisher (which is going to the wholesale when you ought to go to the retail place of business), the address is Messrs. J. and A. Churchill, 11, New Burlington Street, London.

*F. H.*—The offer was to any nurse or institution; we are obliged to confine our "Wants and Workers" to those whose connection with some body gives proof that they are *bona fide*.

*Nurse Emily.*—Your letter has been sent on to the chaplain, and he will accede to your request in due course.

*M. B.*—London Fever Hospital, all the fever hospitals of the Metropolitan Board, Bradford Fever Hospital, Newcastle City Hospital, Leeds Borough Hospital.

*B. B.*—Much regret delay in inserting your query.

*A. L.*—Your only chance would be a provincial hospital, or one badly in need of a nurse. Your age precludes you under the usual rules. Your best chance would be to answer advertisements appearing in our pages. The Red Cross Institution is at Dublin. Pardon delay in answering your queries.

*Cape Colony.*—We beg to inform "Anxious," "J. B.," "Nurse A. M. R.," and others that we will answer their queries next week in an article.

### An Urgent Case.

DR. TRENT was undoubtedly a very hard-worked man. He resided in a country town in that most beautiful of all English counties—Cornwall, and his heart was so big and generous that his purse was seldom full. "A labourer is worthy of his hire," he would say, smiling. "That is a true saying, but how the Dickens can I take a fee from poor folks who earn their bread by the sweat of their brow?"

"Don't say *sweat*, dear," interrupted his sister, Miss Mary Anne, "it is *such* a vulgar word."

Dr. Trent, knowing of no other equally expressive, held his peace.

In sunshine or storm he went on his rounds, and his keen, clear eyes looked through the patient's outer man as though they had power to pierce substantial flesh and bone, and penetrate to the wonderful internal machinery whose intricate workings are so apt to get out of order. Folks might ail or be racked by cruel pains, live or die as the case might be, but Dr. Trent's earnest face still wore the same attentive look, though at times the gentle smile failed him, and the colour fled from his cheek.

This was especially to be remarked one winter evening when he returned somewhat late from a particularly serious case, and he sighed as he flung himself into the old horsehair armchair.

"Dead beat!" grunted Miss Mary Anne.

"Just a trifle weary," replied her brother, apologetically, changing the sigh into a little embarrassed cough.

"And all for nothing! When do you expect to get your fee from those Simpsons I should like to know?"

"Never, my dear!" rejoined the doctor, drily, "seeing that Simpson will die in spite of my efforts, and his widow—aye, there's the rub—we shall have to get up a collection for *her*, poor soul."

Miss Mary Anne snorted, then, laying down her work, broke into speech.

She was so fluent that Dr. Trent became suddenly helpless.

"I believe I *am* tired, after all," said he, "and though it is only ten o'clock, if you will excuse me I will turn in. Let's see, what time did I go to bed last night?"

"You did not go to bed at all," snapped his sister; "what a memory you have. Didn't you sit up all night with old Goody Harris?"

"Bless my soul, so I did; I quite forgot. Well, all the more reason to get a rest now. Good night, sister."

And the doctor, lighting his candle, went slowly upstairs.

"I shall sleep without rocking," observed he; and as soon as his head was on the pillow he fell into a pleasant doze.

(*To be continued.*)

### A Holiday Amusement.

A NURSE, who must surely be having a holiday just now, sends us the following "Four-line Tragedies," which have been composed by a party of frivolous people on a wet day:—

Healthy workman,
Evil smell,
Typhoid fever,
Tolling bell.

Little girl,
Box of paints,
Sucked the brush,
Joined the saints.

Little boy,
Pair of skates,
Broken ice,
Golden gates.

Happy children,
Loaded gun,
Loud explosion,
No more fun.

### Appointments.

[It is requested that successful candidates will send a copy of their applications and testimonials, with date of election, to THE EDITOR, The Lodge, Porchester Square, W.]

CAPE COLONY.—A Nurses' Home and Cottage Hospital has been opened at King Williamstown, Cape Colony. Miss Janet Hickman, of the Nottingham General Hospital, has accepted the post of Matron, at a salary of £80. There is only a small hospital at King Williamstown, entirely for natives, and it is hoped that the new Cottage Hospital will be the beginning of a large and useful Institution. The Home has been started chiefly by the King Williamstown doctors, who are much interested in its success.

MARSDEN, Yorkshire, is starting a district nurse. The post has been offered to and accepted by Nurse Dale of the Huddersfield Nurses' Home.

RIO TINTO.—We hear that nurse S. M. Ferrier, assistant staff nurse in Mr. A. G. Miller's wards, Royal Infirmary, Edinburgh, is about to proceed to Rio Tinto, Spain, having been appointed Head Nurse to the English Hospital there. Nurse Ferrier will be much missed from her place in the Royal Infirmary where she is a general favourite among her patients and sister nurses.

ST. JOHN'S HOSPITAL.—Miss Ada Fuller has been appointed nurse at this hospital at Twickenham, in place of Miss Eva Elliott, who resigns after five years' good work.

### Christmas Competitions.

WE want none of our readers to go away for their holidays without taking some piece of work for wet days, which, when finished, can be sent to us for our Christmas parcels. So heartily were the garments for adults which we distributed last year appreciated, that we want this year to have twice the number. To encourage all to help us in this way, and to add interest to the work, we offer the following prizes, which will be awarded in books or money as the winners choose: (1) For the best pair of socks knitted by a nurse, 5s.; (2) for the best pair of socks knitted by any HOSPITAL reader, 5s.; (3) for the best made flannel shirt, 10s.; (4) for the best made woman's blouse, 10s.; (5) for the best made flannel petticoat, 10s.; (6) for the best made and best shaped dressing gown for an invalid cut out and made by a nurse, 20s. It will be seen that No. 1 and 6 are reserved for nurses only. With regard to No. 6 we specially hope for many entries, and if we secure them we propose to give more than one prize. Flannellette is cheap, and light, and warm, and would, therefore, form the best material for the dressing gown. In judging, four marks are given for workmanship, four for shape, and two for general appearance; therefore, it is not wise to spend time on elaborate trimmings. Long seams may be done by machine.

### Amusements and Relaxation.

SPECIAL NOTICE TO CORRESPONDENTS.

**Third Quarterly Word Competition commenced July 4th, 1891, ends September 26th, 1891.**

Competitors can enter for *all* quarterly competitions, but no competitor can take more than *one first prize* or *two* prizes of any kind during the year.

The word for dissection for this, the FOURTH week of the quarter, being

"DIPLOMAS."

| Names. | July 16th. | Totals. | Names. | July 16th. | Totals. |
|---|---|---|---|---|---|
| Paignton | 29 | 78 | Christie | — | 44 |
| Psyche | 32 | 80 | Dulcamara | 29 | 73 |
| Hope | — | 47 | Nurse J. S. | 34 | 77 |
| Lightowlers | 33 | 80 | Qu'appelle | 34 | 76 |
| Wizard | 28 | 75 | E. M. S. | 28 | 68 |
| Wyameris | — | 46 | Jenny Wren | 28 | 66 |
| Dove | — | 46 | Carpendium | 28 | 65 |
| Punch | 28 | 74 | Grannie | — | 36 |
| Ivanhoe | 32 | 77 | Nurse G. P. | 23 | 58 |
| Tinie | 32 | 77 | Goodnight | 17 | 41 |
| Agamemnon | 33 | 78 | Gamp | 20 | 20 |
| Nurse Ellen | 27 | 71 | Charity | 23 | 23 |

N.B.—Each paper must be signed by the author with his or her real name and address. A *nom de plume* may be added if the writer does not desire to be referred to by us by his real name. In the case of all prize-winners, however, the real name and address will be published.

All letters referring to this page which do not arrive at 140, Strand, London, W.C., by the *first post on Thursdays*, and are not addressed PRIZE EDITOR, will in future be disqualified and disregarded.

## Figures and Tables

**Figure f1:**
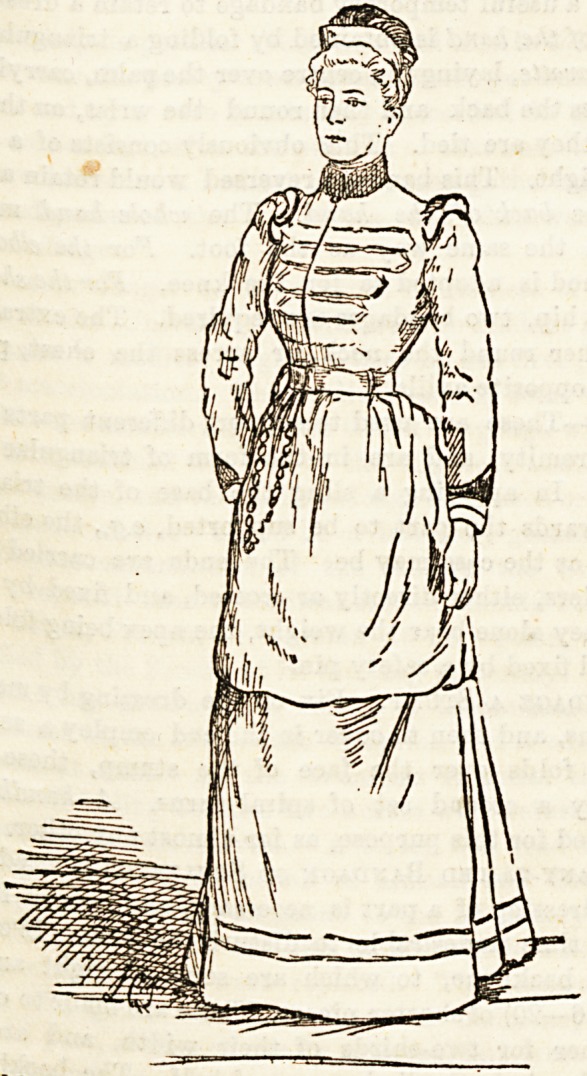


**Figure f2:**
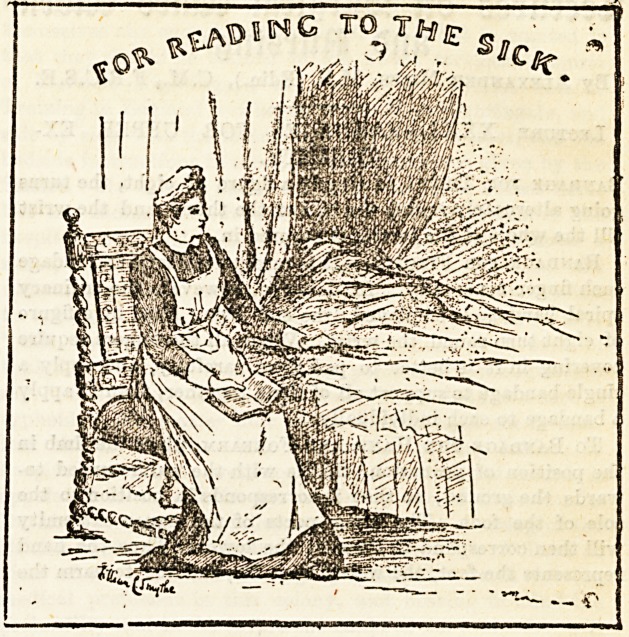


**Figure f3:**